# A Bibliometric Analysis and Benchmark of Machine Learning and AutoML in Crash Severity Prediction: The Case Study of Three Colombian Cities

**DOI:** 10.3390/s21248401

**Published:** 2021-12-16

**Authors:** Juan S. Angarita-Zapata, Gina Maestre-Gongora, Jenny Fajardo Calderín

**Affiliations:** 1 DeustoTech, Faculty of Engineering, University of Deusto, 48007 Bilbao, Spain; fajardo.jenny@deusto.es; 2 Faculty of Engineering, Universidad Cooperativa de Colombia, Medellín 050012, Colombia; gina.maestre@campusucc.edu.co

**Keywords:** crash severity prediction, supervised learning, machine learning, automated machine learning, intelligent transportation systems, Internet of Things

## Abstract

Traffic accidents are of worldwide concern, as they are one of the leading causes of death globally. One policy designed to cope with them is the design and deployment of road safety systems. These aim to predict crashes based on historical records, provided by new Internet of Things (IoT) technologies, to enhance traffic flow management and promote safer roads. Increasing data availability has helped machine learning (ML) to address the prediction of crashes and their severity. The literature reports numerous contributions regarding survey papers, experimental comparisons of various techniques, and the design of new methods at the point where crash severity prediction (CSP) and ML converge. Despite such progress, and as far as we know, there are no comprehensive research articles that theoretically and practically approach the model selection problem (MSP) in CSP. Thus, this paper introduces a bibliometric analysis and experimental benchmark of ML and automated machine learning (AutoML) as a suitable approach to automatically address the MSP in CSP. Firstly, 2318 bibliographic references were consulted to identify relevant authors, trending topics, keywords evolution, and the most common ML methods used in related-case studies, which revealed an opportunity for the use AutoML in the transportation field. Then, we compared AutoML (AutoGluon, Auto-sklearn, TPOT) and ML (CatBoost, Decision Tree, Extra Trees, Gradient Boosting, Gaussian Naive Bayes, Light Gradient Boosting Machine, Random Forest) methods in three case studies using open data portals belonging to the cities of Medellín, Bogotá, and Bucaramanga in Colombia. Our experimentation reveals that AutoGluon and CatBoost are competitive and robust ML approaches to deal with various CSP problems. In addition, we concluded that general-purpose AutoML effectively supports the MSP in CSP without developing domain-focused AutoML methods for this supervised learning problem. Finally, based on the results obtained, we introduce challenges and research opportunities that the community should explore to enhance the contributions that ML and AutoML can bring to CSP and other transportation areas.

## 1. Introduction

One worldwide challenge is designing and promoting policies to reduce traffic crashes, which are one of the leading causes of death and injuries worldwide [1]. In this sense, Intelligent Transportation Systems (ITS) and new Information and Communication Technologies (ICTs) (e.g., the Internet of Things) are crucial factors that can contribute to accomplishing such an aim [2,3]. The synergies between these two technological approaches constantly sense and generate large volumes of data, leading the way towards the design and deployment of data-driven road safety systems (RSS). RSS aims to predict crashes and classify their severity, which is valuable information for traffic managers and policy-makers to increase road safety.

Such data availability, provided by diverse ITS infrastructures (e.g., automatic vehicle identification, cameras), and growing computational capabilities have increased the use of machine learning (ML) to predict crashes and their severity. The main strength of ML, with respect to traffic theory models, is its ability to predict traffic crashes and their severity using current and historical data without knowing the theoretical mechanisms of traffic. From an ML perspective, the crash severity prediction (CSP) problem is focused on building a predictive model using historical data to predict accident severity based on new and unseen data [4].

Transportation literature reports a great variety of ML methods used for CSP (e.g., random forest, neural networks, support vector machines). As a result, there are review papers that organize and systematize the existing knowledge of CSP approaches from an ML perspective. The main contribution of these studies is [4,5,6,7] was presenting state-of-the-art ML techniques for road accident analysis and forecast. However, despite the availability of these methods, there is no unique ML algorithm that is competitive in all kinds of traffic crash scenarios, as stated by the no free lunch theorem [8]. Therefore, deciding on the most suitable ML method for a particular CSP problem is a complex task requiring expert ML knowledge, time, human effort, and high computational capabilities, which are also assets that are not always available.

Automated machine learning (AutoML) arises as a promising strategy to reduce human effort and the time cost of ML in research areas wherein specialized ML knowledge is an asset that is not always available or affordable, such as the case of RSS. AutoML aims at automatically finding competitive ML pipelines (the combination of preprocessing techniques and an ML algorithm) that maximize a performance metric on given data without being specialized in the problem domain from which the data are derived (general-purpose AutoML) [9]. AutoML methods have been successfully used in other areas of the transportation domain, like traffic forecasting [10,11,12,13]. Nevertheless, the extent to which general-purpose AutoML can be competitive in diverse transportation problems, such as CSP, is far from being fully answered. Therefore, it is necessary to identify the strengths and weaknesses of general purpose AutoML versus ad hoc ML methods for CSP to determine whether or not it is worth developing domain-focused AutoML methods to support the model selection problem in CSP.

In this paper, our objective is to continue exploring the line of research proposed in [14] through the development of a bibliometric study and a benchmark of ML approaches for CSP. Specifically, we aim (1) to characterize the performance of diverse ML approaches in various CSP problems experimentally; (2) to compare the competitiveness and the significance of general-purpose AutoML versus *ad hoc* ML methods identified via the bibliometric analysis, and (3) to test whether or not it would be necessary to develop domain-centered AutoML approaches to support the model selection problem in CSP. To accomplish such an aim, we test AutoML (AutoGluon [15], Auto-sklearn [16], TPOT [17]) and ML (CatBoost, Decision Tree, Extra Trees, Gradient Boosting, Gaussian Naive Bayes, Light Gradient Boosting Machine, Random Forest) methods in diverse CSP scenarios using real data from the cities of Medellín, Bogotá, and Bucaramanga in Colombia. In this context, the main contributions of this work are:

- A bibliometric study of ML in CSP. This systematic approach makes it possible to identify relevant authors in the field, trending topics, keyword evolution, and the most common ML methods used in CSP. Although there are already survey papers in the specialized literature [4,5], we expand their scope to the point of identifying and processing 2318 bibliographic references that allow us to characterize state-of-the-art of and AutoML methods in CSP from a theoretical perspective.
- An extensive experimental framework of state-of-the-art ML approaches for CSP. This practical benchmark experimentally characterizes the competitiveness and the significance of diverse ML methods in multiple crash accident settings modeled as supervised learning problems (binary and multiclass classification). This benchmark also provides transportation practitioners and researchers with a framework that supports the use of ML and AutoML in a field where ML expertise is an asset that is not always affordable or available.

The rest of this paper is structured as follows. Section 2 introduces some background information on AutoML and related work regarding previous contributions that are focused on the point where CSP and ML converge. Section 3 introduces a bibliometric study covering the point where ML, AutoML, and CSP converge. Then, Section 4 contains the benchmark of ML-based approaches in supervised CSP problems. Later on, Section 5 summarizes the main results obtained in the benchmark’s experimentation. Lastly, Section 6 summarizes the main conclusions of this research article and sets a research agenda for ML and AutoML in CSP.

## 2. Background and Related Work

This section provides background information on AutoML and related work focused on other literature reviews and experimental comparisons at the point where CSP and ML meet. We start by giving a brief introduction to AutoML in Section 2.1. This section is also complemented with a summary of the main AutoML methods considered in this article. Finally, Section 2.2 reviews the scientific literature that studies CSP from either a theoretical or experimental ML-based perspective. Besides, this section highlights other ML approaches that, although outside the scope of this article, are gaining momentum as potential approaches to CSP, such as deep learning.

### 2.1. Automated Machine Learning

ML is a field that is focused on algorithms that are capable of carrying out prediction/classification tasks by means of an automatic learning process, without the need to be explicitly programmed by human intervention [18,19]. Applications such as e-mail spam and malware filtering, automatic speech recognition, predictive maintenance in industry, among others are based on ML. As a common denominator, all these applications require well-designed and efficient ML pipelines that include data preprocessing as a key factor [20,21].

According to [22], a ML pipeline *P* can be defined as a combination of algorithms *A* that transforms input data *X* into target values *Y*. Wherein *A* is composed of $A_{preprocessing}$, a subset of preprocessing techniques, $A_{feature}$, which is a subset of feature engineering methods, and $A_{algorithm}$, an ML algorithm with a configuration of hyperparameters $\lambda^{i}\in \Lambda$. To build an ML pipeline with this structure, human effort and high computational capacities are needed because no pipeline can perform well in all learning problems [23,24]. This is usually done by data scientists who use their specialized knowledge or non-expert users who tackle the problem via a trial and error approach.

AutoML is an emerging area that automatically finds the best combination of preprocessing techniques and/or ML algorithm and its hyperparameters. The objective is to improve a specific performance metric on a given dataset without being specialized in the problem domain fro which this data is derived [9]. Hence, AutoML reduces human bias and improves computational costs by making the construction of ML applications more efficient. The current literature [9,22,23] reports a variety of AutoML approaches. Within the most representative methods, we can find Auto-Sklearn [16], TPOT [17], and AutoGluon [15]. These are the AutoML methods considered in this paper, and a short description of them is presented below.

- AutoGluon: It starts training its base of classifiers in the usual manner. Then, a stacker model is trained using the aggregated predictions of the base models as its features. Here, the first layer has multiple base models, whose outputs are concatenated and then fed into the next layer, which consists of multiple stacker models.
  These stackers then act as base models for an additional layer. The aforementioned process is repeated multiple times to get a multi-layer stacking approach that improves the shortcomings of the individual base predictions and takes advantage of the interactions between them. Finally, the last stacking layer applies ensemble selection to aggregate the stacker models’ predictions in a weighted manner.
- Auto-sklearn: In an off-line phase, Bayesian optimization is used to determine an optimised ML pipeline with high performance on every dataset for a repository of 121 datasets. These pipelines are generated from a search space of 15 classifiers, 14 feature preprocessing methods, and four data preprocessing methods. Then, for each dataset, a set of 38 meta-features is extracted to characterise every set of data; these meta-features include simple, information-theoretic and statistical information, such as statistics, about the number of data points, the number of classes, and data skewness, among others. Later on, instead of storing the 121 datasets, their meta-features and the ML pipelines are saved in a meta-knowledge base where each instance contains the set of meta-features describing every data-set and the optimized pipeline that works well on it.
  In the online phase, that is, when a new data-set $D_{new}$ is given, Auto-Sklearn computes its meta-features, ranks all the dataset information stored in the meta-knowledge base according to their L1 distance with respect to $D_{new}$, and selects the stored ML pipelines for the *k* nearest datasets. This selection of K most promising pipelines is then used then to seed the Bayesian optimization component as a warm-start approach, which boosts the performance of the optimization. The Bayesian optimization process (under a time budget constraint) also generates and tests new pipeline structures from the same aforementioned search space. Finally, the best pipelines identified during the Bayesian search process are used to construct an ensemble.
- TPOT: To combine these operators into a ML pipeline, TPOT treats them as GP primitives and constructs GP trees from them. To automatically generate and optimize these tree-based pipelines, TPOT uses a genetic algorithm that follows a standard GP process. First, the GP algorithm generates 100 random tree-based pipelines and evaluates their balanced cross-validation accuracy on the dataset. For every generation of the GP algorithm, TPOT selects the top 20 pipelines in the population trying to maximize the classification accuracy and minimize the number of operators in the pipeline at the same time.
  Each of the top 20 selected pipelines produces five copies (i.e., offspring) in the next generation’s population, 5% of these offspring are crossed with another offspring using one-point crossover. Then 90% of the remaining unaffected offspring are randomly changed using a point, insert, or shrink mutation. In every generation, the algorithm updates a Pareto front of the non-dominated solutions discovered at any point in the GP run. The algorithm repeats this evaluate-select-crossover-mutate process for 100 generations to improve classification accuracy. If the user does not define an execution time for TPOT, the method selects the pipeline with the highest-accuracy from the Pareto front as the best representative pipeline of the whole optimization process.

### 2.2. Related Work

Although there is abundant literature in CSP from a ML perspective, not many research articles address the review of ML contributions to CSP or the characterization of ML methods through experimental benchmarks in various CSP problems. Therefore, the most relevant studies are presented below with a brief description of their main contributions.

In 2015, Gajendran et al. [7] reviewed three promising methods to approach CSP. Specifically, they discussed the strengths and drawbacks of System dynamic models, Fuzzy logic, and Bayesian methods in term of the accident severity problem. In addition, the authors highlighted the methodological assumptions of these methods and the data they can use to deal with CSP in different cities in India. Two years later, in 2017, Iranitalab and Khattakb [25] compared the performance of four statistical and ML methods in CSP. The methods included Multinomial Logit, Nearest Neighbor Classification (NNC), support vector machines, and random forests. The authors used real data from Nebraska, United States, and demonstrated that NNC had the best prediction performance overall.

Later on, in 2019, Tang et al. [26] introduced a two-layer stacking method (composed of random forests, AdaBoost, and gradient boosting decision tree, and logistc regression) to predict crash injury severity. The performance of the stacking model was compared with support vector machine, multi-layer perceptron, and random forest. The prediction results show that the stacking model achieves superior performance by putting together competitive ML methods that outperform individual techniques. In addition, although this paper is not a benchmark *per se*, it established an interesting baseline for ML techniques to make comparisons in CSP.

In 2020, Tang et al. [6] designed and developed a methodology review for statistical and ML methods to cope with clearance time prediction of road incidents, which is a transportation problem that is closely related to CSP. The authors considered four widely used statistical models: accelerated failure time model, quantile regression model, finite mixture model, and random parameters hazard-based duration model; and four ML methods: K-nearest neighbor, support vector machine, back propagation neural network, and random forest. The authors concluded that ML methods, especially random forest, have a more stable performance than statistical methods.

In the same year as [6], Gutierrez-Osorio et al. [5] reviewed papers that used ML methods for CSP. The authors identified three instances of CSP: crash frequency prediction, crash classification by severity, and crash frequency and severity prediction. These problems are usually modeled as supervised regression, supervised classification, and a mixture of these two ML approaches, respectively. Additionally, the authors reviewed the performance metrics usually considered when making comparisons between methods.

Complementarily, in 2020, Silva et al. [4] provided an overview of state-of-the-art in road accident prediction using ML algorithms, including more complex methods such as deep learning (e.g., convolutional neural networks, long short-term memory networks). In this regard, although deep learning is beyond the scope of this research article, we emphasize that the contributions of this approach to the field of CSP are far from complete. The latest contribution is supported by some recent advances reported in the scientific literature, such as the cases in [27,28,29,30]. These authors show how the whole road safety field (including CSP, collision warning systems [31], causality in highway-safety [32]) can benefit from deep learning methods.

In summary, we can say that the literature reports well-established contributions regarding survey papers, experimental comparisons among various ML-based methods, and the design of new and novel methods at the point where CSP and ML converge. In this sense, and taking the literature previously presented as starting point, we complement the state-of-the-art in the following ways. First, we design and present a bibliometric study and an extensive experimental framework of state-of-the-art ML methods in CSP. This seeks to consolidate previous theoretical and practical contributions reported in the literature in a single research effort, which supports the model selection in CSP. Second, we incorporate AutoML into the CSP field, which is a state-of-the-art data-driven approach in multiple problem domains that are not limited to the field of transportation. This will provide interesting insights into how this automated ML approach can contribute to the field of road safety, where expert ML knowledge is an asset that is not always affordable or available. Finally, we experimentally assess the performance of ML and AutoML in various crash severity scenarios modeled as binary and multiclass classification problems. These scenarios are represented by real data from three cities, which allows the suitability and competitiveness of AutoML to be tested in diverse CSP scenarios.

## 3. Bibliometric Analysis of Machine Learning Approaches in Crash Severity Prediction

The main objective of this bibliometric analysis is to investigate the current state of research at the point where CSP and ML converge. This study is a systematic and analytical technique supported by specialized software for data capture and analysis, which allows relevant authors, their affiliations, keywords, and trends related to the study area to be identified [33]. Furthermore, the bibliometric approach is appropriate when assessing the current state of a given discipline by means off a transparent, static, and systematic representation of research [34]. Therefore, this study relies on bibliometric analysis as an ideal method to examine the current knowledge base of applications in three topics: (1) ML for road accidents, (2) crash severity forecasting and, (3) AutoML in CSP and transportation.

**Figure 1:**
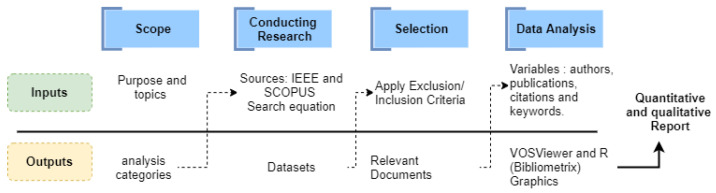
Protocol followed for the bibliometric analysis carried out in this study.

The bibliometric analysis used protocol Figure 1 defined below, which begins with the definition of the purpose and the selection of the topics to be analyzed. Then, there are three sequential steps that direct the search strategy, the inclusion and selection criteria, and the analyses supported by specialized software that facilitate the reporting of results. Bibliometric indicators were generated using R and WoSViewer. We defined the following analysis categories: authors, sources, documents, keywords, time evolution of the topics, and techniques used in the study cases.

### 3.1. Conducting Research

This study utilized the SCOPUS and IEEE databases for data collection and screening. SCOPUS and IEEE are world-leading scientific databases widely known for their extensive, reliable content and many publications [35]. Besides, IEEE is the most relevant and specialized database on engineering topics, with the most influential journals and conferences in computer science and transportation.

The search was performed in the title, abstract, and keywords fields for each topic, and the search string used for IEEE and SCOPUS was the following: Title-Abs-Key ((road AND accident) AND (machine AND learning)); Title-Abs-Key ((machine AND learning OR ml) AND (crash AND prediction OR crash AND forecasting OR crash AND severity AND forecasting OR crash AND severity AND prediction)); Title-Abs-Key (“automl” OR “automated machine learning” OR “automatic machine learning” OR “auto machine learning” OR “Auto ML”).

### 3.2. Selection

**Figure 2:**
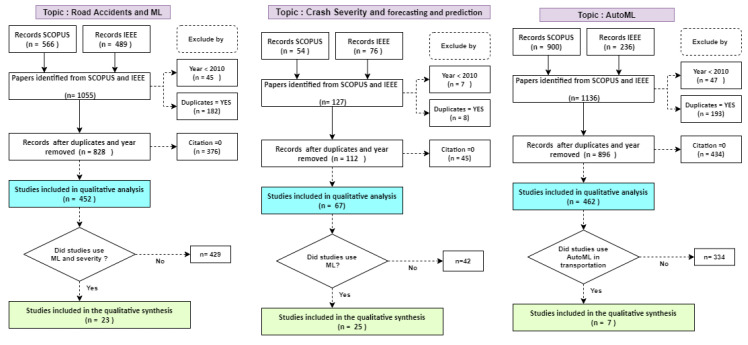
Criteria considered for the inclusion and exclusion of literature related to CSP, ML, and AutoML.

In this stage, the inclusion and exclusion criteria are applied as shown in Figure 2. The steps followed are explained as follows: (1) a single dataset is generated for each topic of interest with the records obtained from the IEEE and SCOPUS databases with books, journals, and conferences publications; (2) eliminate records with publication year < 2010; and (3) eliminate duplicates; then, (4) exclude documents with 0 citations to select the most influential publications in the research fields of interest, following the guidelines introduced by [36,37] for bibliometric analysis; finally (5) include studies according to the relevance of title and abstract.

For road accidents and ML, 1055 records were initially considered, 452 were finally selected for quantitative analysis, and 23 were chosen for qualitative analysis as shown in Figure 2. For Accident Severity and forecasting, 127 records were obtained, of which 67 were selected for quantitative analysis and 25 for qualitative analysis. Finally, for the AutoML topic, 1136 records were obtained, 462 studies were analyzed, and 7 studies were selected because they were related to transportation. The details of the selected dataset can be seen in Table 1 (datasets are available at this repository (https://drive.google.com/drive/folders/1JEyGlonRf2Nj2YJ2bIZrGO18Jy6VvMjf?usp=sharing, accessed on 29 October 2021)).

### 3.3. Data Analysis

Two types of analysis have been considered as shown in Figure 2. First, in the quantitative analysis, R and WosViewer software was used to generate the analyses related to authors, trends, the evolution of keywords, and the most relevant publications for each topic. Second, regarding the qualitative analysis, 48 studies focused on crash severity and road accident topics were selected to be analyzed. Based on references, we have defined the application contexts and ML methods applied. In the case of AutoML, no study was taken as a reference because no applications in road accidents or crash severity were found.

**Figure 3:**
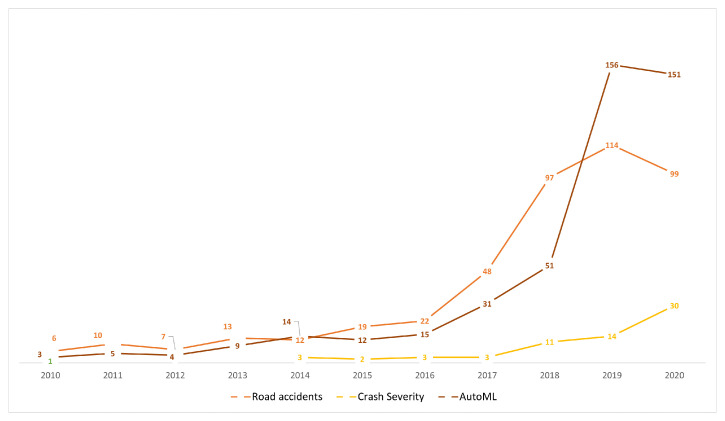
Year-wise distribution of research in the fields of Road Accident, Crash Severity, and AutoML.

**Figure 4:**
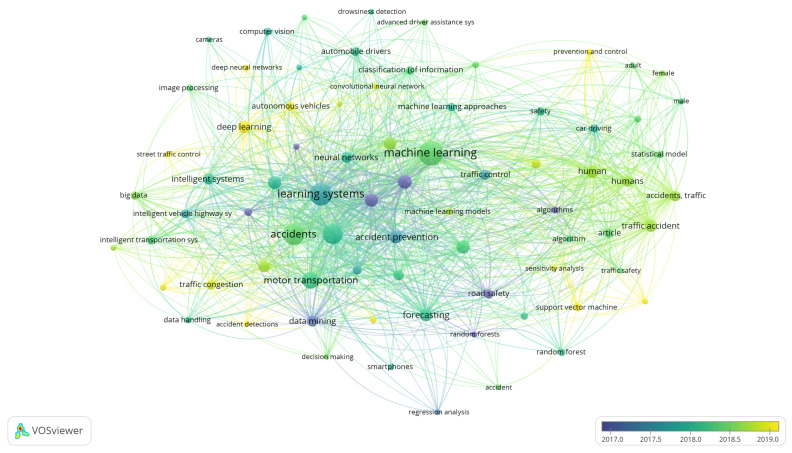
Keywords related to road accidents approached from a ML perspective.

**Figure 5:**
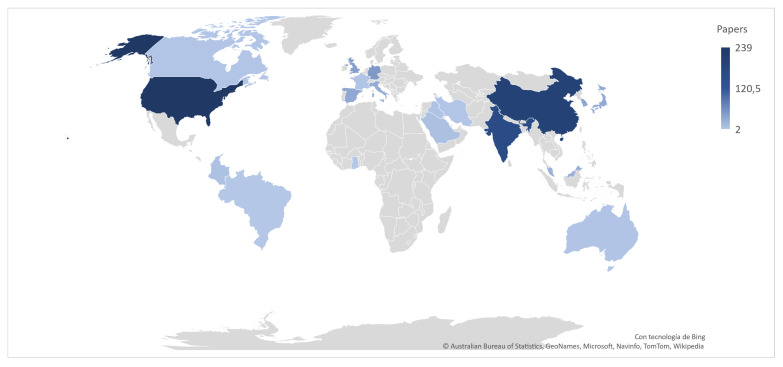
Countries with the highest scientific production at the point where CSP and ML converge.

- **Time trend of publications:** Since 2014 there has been a growing trend of publications related to ML, AutoML, crash severity and road accidents, which is reflected in the number of annual publications. This shows the relevance and timeliness of these fields for conceptual, methodological, and practical applications (Figure 3). Moreover, in the transportation field, road accidents show a growing trend, and since 2014 the specific topic of the severity of collisions has been emerging rapidly. This is supported by the fact that in 2020 there was a significant scientific production in this regard, which was mainly focused on a case analysis of cities using various modeling tools, simulation, and machine learning approaches.
  When analyzing the three topics selected for this paper, we can highlight that the average number of citations per document for CSP is 18.51, 9.66 for road accidents, and 10.31 for AutoML. Together with the increasing trend in the number of publications, this is indicative of the interest related to crash severity issues. There has been significant scientific production concerning the topic of AutoML over the last 5 years; however, its application is focused on concentrated in health, and there are few works on the topic of transportation. Furthermore, most of the applications in this field are oriented towards traffic prediction [10,11,38], transport modes [39], and autonomous vehicles [40]. Thus, no applications focused on CSP were found in the review.
- **Relevant sources, documents and authors in the field:** The most influential journals in the study topics are: accident analysis and prevention, IEEE transactions oniIntelligent transportation systems, and transportation research record. All journals are focused on transportation and safety issues. On the other hand, IEEE Access stands out with a focus on applications, for example, computational applications in engineering, as shown Table 2.
  The most influential authors (Table 3) include Abdel Aty with his study on accident and traffic modeling and injury severity [41,42], and Lee J and Mannering with their articles on impact and accident assessment [43,44]. In the topic of severity analysis by prediction with ML, the works of Li [45,46] and Zhang [47] are the most relevant papers. The top 10 most globally cited articles present in Table 3 include: Sivaraman et al. [48] who explain a new active learning approach to developing vehicle recognition and tracking systems. Martinez et al. [49] present a study on driving style characterization and recognition by reviewing various machine learning-oriented algorithms. Desjardins et al. [50] propose a new concept for the design of autonomous vehicle controllers based on advanced ML techniques. Zhu [51] present a framework for big data analytics in Intelligent Transport Systems. Meiring et al. [52] analyze the applicability of ML and artificial intelligence algorithms to predict the driver’s behavior and driving style. Young et al. [53] explore the types of data used to develop, calibrate and validate CSP models. Finally, Ji et al. [54] explore the predictive potential of variables related to the collision mechanism using ensemble learning models.
- **Evolution of keywords and topics:** Analyzing keyword trends, Figure 4 shows that learning systems, especially ML, strongly influence applications directed to accident prevention policies. In recent years, terms related to the integrity of people and safety related to the severity of accidents have gained relevance. ML techniques are essential for modeling accident-related phenomena, mainly because of structured data available in open access repositories. Figure 4 also shows that new methods are emerging to address CSP, especially deep learning. This is due to the availability in recent years of open and unstructured data, mainly spatial, images or GPS data, which facilitate the use of these emerging methods.
- **Machine Learning Methods:** About 13 studies in the last four years were selected for analysis. In Table 4 we can observe that the most frequent methods for CSP are random forest (RF), used in 7 of the 13 studies, decision tree (DT) in 6 papers, support vector machine (SVM) in 5 articles, and Naive Bayesian (NB) in 4 studies. These methods are commonly used under the supervised learning paradigm, where CSP is modeled as a classification problem. On the other hand, we identified the following methods when analyzing the ML approaches that show the best performance in the studies consulted. RF [56,57,58], SVM [59,60,61], Light-GBM [62], Gradient Boosting [63], AdaBoost [58], Multi-layer perceptron [64], Nearest Neighbor Classification [25], and SimpleCart model [65]. These methods were commonly used for case studies located in the USA (Connecticut, Michigan, California, Florida, Nebraska), United Kingdom, China (Kunshan), Korea (Seoul), India, and Ghana. Thus, these geographic areas contain the majority of the scientific production on these topics, as shown in Figure 5.

## 4. The Proposed Benchmark

This practical section aims to provide experimental insights into the model selection problem in CSP. To accomplish such an aim, we compare the performance of diverse ML methods in three case studies modeled as supervised classification problems (binary and multiclass problems). The selection of the ML methods is guided by the results obtained in the bibliometric analysis shown in the previous section. Thus, we chose the methods that, according to the literature, demonstrate a robust performance across the different studies consulted. In addition, we also test whether general-purpose AutoML can be competitive against these *ad hoc* ML methods in the three case studies.

The following parts of this section are devoted to presenting the case studies and their raw data in Section 4.1. Next, Section 4.2 introduces the datasets that were generated from the raw data for the experimentation. Then Section 4.3 shows the experimental set-up proposed, which includes the performance metrics, the ML methods for the experimentation, and the statistical tests selected to make comparisons among the ML methods.

### 4.1. Case Studies and Raw Data

Colombia has approximately 49 million inhabitants. In 2020, it reported 16,042,336 registered vehicles, of which 59% are motorcycles, while vehicles such as trucks, vans, buses, vans, and mopeds represent 40%. Private vehicles are the most relevant in Colombia, at 92%, while public services represent 6%, and other types of services represent the 2% [67].

Road safety in Colombia is a serious public health problem and is the second leading cause of death. According to a report by the Institute of Legal Medicine, in 2019, nearly 7000 people lost their lives in road accidents, and more than 35,000 people suffered severe injuries. The primary victims are motorcyclists (53%) and pedestrians (21.8%), followed by motor vehicle users (12%) and bicycle users (8%). This phenomenon impacts the Colombian economy with 23.9 trillion pesos annually, which is equivalent to 3.6% of the Gross Domestic Product [68].

This paper will take three Colombian cities as case studies: Bogota, Medellin, and Bucaramanga. These cities are characterized for having open data portals in the transportation domain. Bogota is the capital of Colombia with approximately 8 million inhabitants, Medellin is the second-largest city in population with 2.5 million inhabitants, and Bucaramanga is a mid-size city with more than 600 thousand inhabitants. It should be noted that although Bogota is the capital country, has the largest population and is the economic center of the country, Medellin is the city with the highest accident rate per population. Bogota and Bucaramanga have an accident rate of 0.004 and 0.006, respectively), and Medellin has a rate of 0.016 [69].

The raw data was obtained from open data portals from each city and correspond to data obtained from different IoT from the integrated transportation system, such as cameras, speed sensors, and online reporting systems of road incidents. These data records are subsequently processed, stored, and made available through local and national open data portals. The details of the raw data are shown in Table 5.

### 4.2. Datasets

We have generated different datasets for each case study from the raw data presented above. Table 6 shows Medellín’s datasets where CSP is approached as a binary classification problem. Each dataset corresponds to 1 year of historical data. Crash severity is the variable to be predicted, and it has two possible labels that characterize the severity of an accident: *people injured* and *only material damages*.

Each dataset contains the same nine attributes, composed of traffic-related and calendar features. Traffic attributes are *Type of accident*, *GPS coordinates of the accident*, and *Type of road where the accident occurs*, whereas calendar features are *Minute*, *Hour*, *Day of the week*, *Day of the year*, and *Month*. In addition, Table 6 shows the imbalance ratio of every dataset, which is calculated by dividing the majority class by the minority class. As can be seen, all five datasets are well-balanced.

Table 7 shows the datasets generated for Bogota (Bog) and Bucaramanga (Buc). As can be seen, these two case studies are modeled as multiclass classification problems. One single dataset corresponds to 1 year of historical data. Crash severity is once again the variable to be predicted, and it has three possible labels that characterize the severity of an accident: *people injured*, *casualties*, and *only material damages*. Table 7 also shows the number of instances and the imbalance ratio per dataset. As can be observed, there are well-balanced and highly imbalanced datasets in both case studies.

Bogota’s datasets have 12 features. Traffic attributes are *ID of the crash accident*, *type of accident*, *Number of casualties*, *Number of people injured*, *Neighborhood*, and *Type of road*. These datasets also have calendar features, which are *Day of the week*, *Day of the year*, *Month*, *Hour*, and *Minute*. In the case of the Bucaramanga case study, the datasets also contain traffic and calendar features. Such attributes are *Type of vehicle involved in the crash*, *Type of road*, *Neighborhood ID*, *Neighborhood name*, *Day of the week*, *Day of the year*, *Month*, *Hour*, and *Minute*.

### 4.3. Experimental Set-Up

In this section we introduce the ML and AutoML methods chosen for the experimentation, the metric used to measure performance, and the statistical tests considered to assess the significance of the results.

- **AutoML and ML methods**: AutoML competitors are AutoGluon (Ag), Auto-Sklearn (As) and TPOT (Tp) with its default hyperparameter values. Ag and As were used for three execution times (15, 60, and 150 min) while Tp did not have an allocated time. Such an execution time corresponds to the time the methods take to find the best ML algorithm and its hyperparameter configuration for a given dataset. The assumption is that longer time budgets lead to better results. Therefore, such a progressive increase from 15 to 150 minutes should exemplify the expected behavior [70]. Additionally, every execution time assigned to a particular AutoML method is considered to be an individual AutoML competitor.
  As baseline methods, we used CatBoost (CatB), Decision Tree (DT), Extra Trees (ExtraT), Gradient Boosting (GB), Gaussian Naive Bayes (GnB), Light Gradient Boosting Machine (LGBM), Random Forest (RF), and a tuned Random Forest (tuned_RF). Moreover, it is relevant to note that we have not performed any optimization or tuning of the hyperparameters of the AutoML methods or the baseline methods. The above is justified because we aim to compare the performance of AutoML versus the baseline using the same human effort for both in order to carry out a fairer comparison.
- **Performance metrics and Statistical tests:** For the results of this paper, we followed the same experimental set-up proposed in the studies by Gijsbers et al. [71] and Angarita et al. [14]. They introduced earlier methodological guidelines to compare AutoML methods, and compare these techniques with other ML methods in CSP, respectively. Specifically, the area under the receiver operator curve (ROC_AUC) is used for the binary classification problems considered in this benchmark. For the multiclass problem, we used the Log Loss function. In addition, the final score achieved by every method is the average.
  We made use of non-parametric statistical tests to assess the differences method performance. Two statistical tests are used following the guidelines proposed in [72]. First, Friedman’s test for multiple comparisons is applied to check whether there are differences among the methods. Then, the Holm’s test is used to check whether the differences in the Friedman ranking are statistically significant or not.

## 5. Results

The experimentation carried out in this benchmark is focused on three objectives. First, to characterize the performance of ML in the crash severity problem. Second, to compare the competitiveness and the significance of general-purpose AutoML versus *ad hoc* ML methods identified in the Bibliometric analysis in binary (Medellín) and multiclass (Bogotá, Bucaramanga) problems with different degrees of imbalanced data. Third, to test if it would be necessary to develop a domain-focused AutoML for CSP to support the model selection problem or if a general-purpose AutoML might be sufficient for such a purpose.

**Figure 6:**
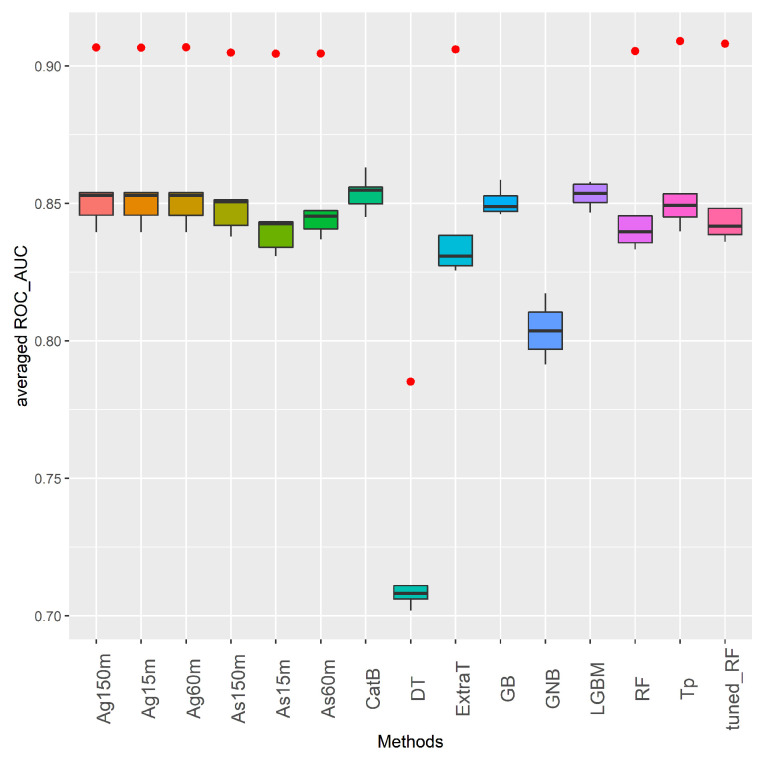
Aggregated ROC_AUC results of ML and AutoML methods in binary datasets from Medellín case study.

Keeping the aims mentioned above in mind, Figure 6 shows the general performance of ML and AutoML methods in the binary Medellín datasets. This figure contains box-plots with information on the distribution of the results of each method obtained from global binary datasets. The X-axis displays the evaluated methods, while the Y-axis shows their performance measured in ROC_AUC score. The performance metric shown for each method is the mean value obtained in the 5 binary datasets after carrying out a 10 cross-validation process per dataset. In the boxes, the central horizontal line indicates the median, the hinges of the boxes indicate the first and third quartiles, and the whiskers indicate the value 1.5 *IQR* where *IQR* is the interquartile range. The red dots below or above each box-plot refer to outlier values.

According to Figure 6 it can be said that all the methods are consistent in dealing with well-balanced binary problems. All methods have high performance in this type of supervised learning problem except for *DT* and *GNB*. Although these two methods perform well above 70%, their ROC_AUC scores are the lowest among the ML and AutoML algorithms. As far as the AutoML methods (*Ag* and *As* with three ETs, and *Tp*) are concerned, their performance is fairly uniform. Specifically, there is no AutoML method that consistently outperforms all the AutoML competitors. This last point is consistent with other AutoML results in transportation problems (supervised traffic forecast and supervised CSP) where longer run times do not necessarily lead to drastically better results [10,11,14].

As for the classic ML methods in Figure 6, it is quite interesting to see how *CatB* and *LGBM* perform quite competitively with respect to AutoML approaches. Indeed, *CatB* is the method with the best performance in all binary datasets. Nevertheless, to conclude that *CatB* and *LGBM* are the best methods in the baseline of ML methods, the user should run all the algorithms on the global baseline datasets. This is where the contributions of AutoML come in, since by deploying any of the AutoML methods, with at least a short execution time and using less human effort, the user can achieve similar or better results than those obtained by *CatB* and *LGBM*.

**Figure 7:**
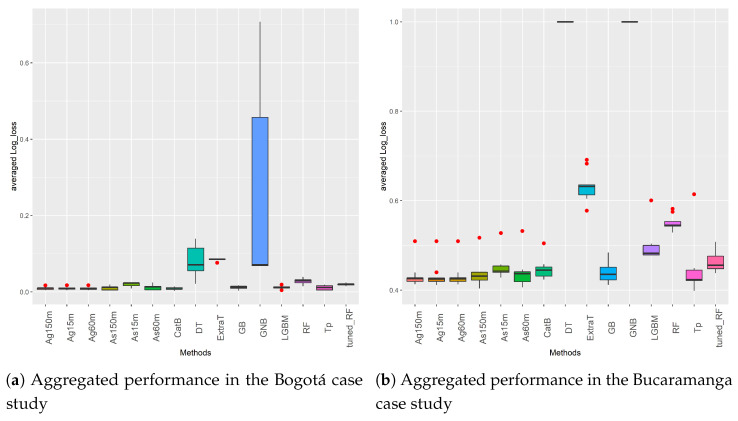
Aggregated log_loss results of ML and AutoML methods in the Bogotá and Bucaramanga multiclass datasets.

Having presented the binary dataset results, Figure 7 summarizes the results obtained in the multiclass problems. This figure shows box-plots with information on the distribution of the results obtained in all multiclass datasets by each ML and AutoML method. The X-axis displays the methods evaluated. The Y-axis shows their performance measured in terms of the log_loss score. The performance metric shown for each method is the mean value obtained in the five Bogota datasets (Figure 7a) and the nine Bucaramanga datasets (Figure 7b) after doing a 10 cross-validation per dataset. Once again, the central horizontal lines of the boxes indicate the median, the hinges of the boxes indicate the first and third quartiles, and the whiskers show the value 1.5 *IQR*.

Figure 7a displays the results obtained from the Bogotá datasets. In this case, all AutoML methods and the ML methods *CatB*, *LGBM*, *GB*, *RF*, and *tuned_RF* perform competitively. It is quite interesting no note that all datasets from this case study have a minority class (Casualties) that is quite unbalanced with respect to the other two classes (Only material damages and People injured). Despite this, the methods mentioned above can deal with this data characteristic effectively. On the other hand, *DT*, *ExtraT*, and *GNB* are the methods with the worst performance and the highest variability in the aggregated results, as shown in Figure 7a.

Figure 7b summarizes the results of the second case study related to multi-class problems. It should be noted that the Bucaramanga datasets have the highest level of imbalance. At the same time, they are also the datasets with fewer instances as compared to data from Medellín and Bogota. These characteristics affect the ML methods’ performance in particular. As can been, they present high variability in the results, and their log_loss score is higher (worse) than that of the AutoML methods. For the case of AutoML, *Ag* and its ETs are the best methods in all Bucaramanga datasets.

Finally, to assess whether the differences in performance observed in Figure 6 and Figure 7 are significant or not, we made use of non-parametric statistical tests. Two statistical tests have been applied following the guidelines proposed in [72]. First, Friedman’s test for multiple comparisons has been used to check significant differences between the ML and AutoML methods. Then, the Holm post-hoc test was also applied to assess the significance of the differences in performance.

Considering that the *p*-value returned by these tests was 0, the null hypothesis can be rejected. Thus, the mean ranking returned by the test is displayed in Table 8. The results in Table 8 confirm the better global results of *Ag* with its three ETs, *Tp*, *As* with 60 and 150 min of ET, *CatB*, and *GB* in the binary and multiclass problems. Holm post-hoc tests used *CatB* and *Ag60m* as control algorithms in binary and multiclass datasets, respectively. This is due to the fact that these two methods got the first position in the average ranking output obtained with the Friedman’s test. Table 8 also presents the adjusted *p*-values returned by this test. In order to highlight significant differences, those *p*-values lower than 0.05 are shown in bold. By looking at Table 8, we can observe that there are important differences in the test’s outcomes. It can be said that *CatB* is statistically better than *GNB* and *DT* in binary problems. Additionally, *Ag60m* is statistically better than *As15m*, *LGBM*, *tuned_RF*, *RF*, *ExtraT*, *DT*, and *GNB*.

## 6. Conclusions

This final section introduces the main reflections drawn from the research carried out in this paper. Section 6.1 introduces the summary and conclusions. Then, Section 6.2 presents a set of challenges and research opportunities to encourage further exploration and use of ML and AutoML in CSP and other transportation-related areas.

### 6.1. Summary

This paper introduced a bibliometric analysis and a benchmark of ML and AutoML in CSP. We aimed to provide a framework that supports the use of ML in a field where expert ML knowledge is an asset that is not always affordable or available. The bibliometric study investigated the current state of research on CSP addressed from a ML perspective. It allowed us to identify typical methods used in different CSP case studies. Then, the ML-based methods identified were used for the baseline of the benchmark to make comparisons with the AutoML approaches.

From the bibliometric analysis, we figured out that scientific literature reports various ML methods (e.g., Random forest, Gradient Boosting, Light Gradient Boosting) that perform competitively in different supervised CSP problems. However, as is well-stated by the *no free lunch theorem*, there is no single ML method that that is competitive in all crash prediction problems. Therefore, practitioners and non-expert ML users must carry out extensive experimentation to determine the most suitable ML method for the particular CSP problems they are addressing. Although this is a strategy that can yield good results, it requires time, computational capabilities, and human effort. From the bibliometric analysis, we also observed the opportunity to analyze and predict CSP using AutoML because it is a topic that has been barely explored in literature. Although there are some AutoML applications in transportation, they are mainly focused on traffic forecasting.

In the context mentioned above, AutoML arises as a suitable approach to automatically address the model selection problem in CSP. Nevertheless, an extensive analysis that determines AutoML’s strengths and weaknesses versus *ad hoc* ML methods has not been carried out in very diverse learning CSP scenarios. Thus, the bibliometric study has been complemented with a benchmark of state-of-the-art AutoML (AutoGluon, Auto-sklearn, TPOT) and ML methods (CatBoost, Decision Tree, Extra Trees, Gradient Boosting, Gaussian Naive Bayes, Light Gradient Boosting Machine, Random Forest). This benchmark aimed: (1) to characterize the performance of ML in CSP addressed from a supervised learning perspective using real data from three case studies; (2) to contrast the competitiveness and significance of general-purpose AutoML versus *ad hoc* ML methods identified in the bibliometric analysis in binary and multiclass problems with different degrees of imbalanced data; (3) to evaluate if it would be necessary to develop specific AutoML approaches for CSP or whether general-purpose AutoML would be sufficient to deal with this supervised learning problem.

From the benchmark’s experimentation, we concluded that most ML methods consistently deal with well-balanced binary problems. In the case of AutoML approaches, their performance is fairly uniform, and it does not improve with longer execution times. Therefore, with a relatively short time, the transportation user can expect competitive results. Regarding the classic ML methods, *CatB* and *LGBM* perform competitively with respect to the AutoML methods. In fact, although there were no significant differences between them, *CatB* was the method located in the first position of Friedman’s average ranking for binary datasets. Thus, it is a suitable alternative to AutoML methods when dealing with CSP binary problems.

On the other hand, with respect to multiclass problems, all AutoML methods, *CatB*, *LGBM*, *GB*, *RF*, and *tuned_RF* perform competitively in the Bogotá case study, which is characterized by having a high degree of imbalanced data. In the Bucaramanga case study, *Ag* and its ETs are the best methods for dealing with the associated datasets, which are the most challenging as they have the highest degree of imbalanced data and the lowest number of instances. This is corroborated by the Friedman’s average ranking that put *Ag15m*, *Ag60m*, and *Ag150m* in the first place in the ranking. Thus, this general-purpose AutoML method is quite competitive and robust in scenarios facing imbalanced data. Consequently, the competitiveness of this AutoML approach can meet the demand of various crash accident scenarios without developing domain-focused AutoML methods.

Finally, we note that the data used in the experimentation present some challenges, such as incomplete data, lack of traceability of the data source, data inconsistency, outdated data, or errors that may limit its use and could affect the performance of ML methods. Despite this, the open data portals, which provided the data used, facilitate access to large amounts of official data obtained from city IoT platforms, which would otherwise require more extensive collection and processing efforts. This allows the development of research studies to be more agile due to data availability in formats that are ready for analysis and processing. Therefore, as future work, we would like to approach CSP problems and other transportation applications in several cities of Colombia, which also have Open Data Portals with traffic data. The goal would be to validate the ML and AutoML methods used in this study and compare case studies with similar and divergent characteristics.

### 6.2. Challenges and Research Opportunities

This section presents challenges and research opportunities that the research community and practitioners should explore in order to improve the contributions that ML and AutoML might bring to RSS, CSP, and other transportation-related areas.

#### 6.2.1. The Method Selection Problem: From AutoML towards Automated Deep Learning

As can be seen in the results obtained via the benchmark analysis (Section 4), different types of ML techniques perform better or worse depending on the CSP problem at hand. These variations on the methods’ performance could be caused by the degree of imbalance in the input data, or the quality of the data, among other aspects. Therefore, these particularities of every CSP problem suggest the need for expert ML knowledge in order to select the best ML workflow (the combination of preprocessing techniques and ML methods). However, this knowledge asset is not always affordable or available for RSS development.

In the above context, AutoML and automated deep learning (AutoDL) [73] are promising strategies to support the role of ML experts and to reduce the human effort and the time cost of ML and deep learning in RSS. Based on the results obtained in this research, we can say that general-purpose AutoML can support the model selection problem in CSP. Nevertheless, we cannot say the same about AutoDL. According to recent literature [27,28,29,30], deep learning is gained great interest due to its ability to address more network-level CSP problems without the need to define preprocessing approaches for the input data. This, therefore, reduces the human effort of using deep learning in CSP. However, finding the most suitable architecture is a demanding task, which consumes the same resources mentioned above: ML knowledge, time, human effort, and computational capabilities.

In this sense, extensive analyses are required to characterize the performance of AutoDL in various CSP and other transportation problems. Particularly, special attention needs to be paid to figure out if general-purpose AutoDL can be competitive in specific problem domains, such as CSP. If that were the case, AutoDL could potentially accelerate the adoption of deep learning in RSS, both from an academic perspective and for RSS deployment.

#### 6.2.2. Data Fusion and Real-Time Data to Enhance the Power of ML and AutoML in CSP

ML and AutoML methods cannot normally incorporate data from different sources. Indeed, in RSS, data coming from a single type of sensor might not be sufficient to represent the complexity underlying CSP problems. For instance, diverse Internet of Things (IoT) devices (e.g., cameras, loop detectors, radars, infrared sensors) provide data with distinct formats to manage RSS (e.g., historical traffic records in the form of tables, non-structured data like images). In this context, the challenge lies in defining guidelines for the harmonization and fusion of data that adequately guarantee the performance of ML and AutoML approaches.

Specifically, the aim is to define suitable guidelines that enable single dataset to be collected and generated with information obtained from different sources. This challenge significantly affects the performance of current AutoML methods, as these approaches are designed to work exclusively with tabular data [9]. Therefore, data from IoT devices like cameras and radar sensors, which contain traffic accident data in the form of images and signals, could not be analyzed and mined under the current design of AutoML methods. Thus, this data fusion challenge also demands the re-design of AutoML techniques to incorporate the diversity of data formats available in RSS.

Data fusion is a challenge that is part of a more significant research opportunity: the management and fusion of real-time data in the context of RSS. Concretely, in supervised learning, the input data is available before starting any training processes. The aim is to train a model from such data using a batch approach. This means that ML and AutoML methods use all available samples in the input data to build and train a model that should work on new and unseen data. Currently, most ML and AutoML applications are focused on the batch learning approach, wherein data is available before training a model [74]. In this context, model training is solely supported on an input dataset whose data distribution does not change over time. Nevertheless, such a static approach is not the case for real CSP applications within RSS and Intelligent Transportation systems.

ML and AutoML must face real CSP scenarios, wherein different IoT devices continuously generate new data streams. Hence, the additional challenge to data fusion is to design of ML and AutoML methods that adapt to real-time data. Further research is needed in the design and deployment of ML and AutoML methods for CSP and related applications, using the concepts of incremental learning [75,76] and data drift detection [77].

#### 6.2.3. Explainability for a Better Crash Severity Understanding

According to the results presented in this paper, ML and AutoML demonstrate their ability to address various CSP problems. However, performance is not the only objective in current RSS and other Intelligent Transportation System domains. The explainability of predictions made by ML and AutoML is also relevant for decision-makers and transportation managers in order to extract relevant risk factors and implement related policies [30].

While ML methods (e.g., rule-based learners, decision trees) can provide different degrees of interpretability of decisions made in the inner structure of the methods, AutoML and AutoDL approaches cannot usually offer this information [78]. In these automated methods, there are few clues regarding the methods’ decisions to obtain high performance. For instance, AutoDL methods are composed of search spaces that contain complex architectures with intricate connections between layers and neurons.

Thus, understanding how AutoML and AutoDL can obtain their competitive performance becomes a relevant and exciting challenge. As AutoML and AutoDL are increasingly used employed in various applications areas [11,79], the demand for explainability and interpretability is growing among various stakeholders, including those involved in RSS and CSP. This request is based on the fact that decisions made based on AutoML, and AutoDL may lead to the formulation of policies that may require detailed explanations [80]. This is known as explainable artificial intelligence (XAI) and is widely acknowledged as a crucial concept to take into account in the understanding of the severity of traffic accidents. As stated in [81], the challenge of XAI in different knowledge domains, such as CSP, is to generate more explainable methods while maintaining their output performance. Hence, research opportunities lie in the design and inclusion of diverse XAI approaches in the AutoML and AutoDL workflows.

## Figures and Tables

**Table 1:** Bibliographic information of the studies reviewed for Road Accident, Crash Severity, and AutoML.

**Main Information**	**Road Accident**	**Crash Severity**	**AutoML**
Timespan	2010:2021	2010:2021	2010:2021
Sources (Journals, Books, etc.)	310	53	333
Documents	452	67	462
Average years from publication	3.21	2.25	2.55
Average citations per documents	9.668	18.51	10.31
Average citations per year per doc	2.134	6.368	2.654
References	10,278	1870	16,000
**Document Types**	**Road Accident**	**Crash Severity**	**AutoML**
Article	194	37	232
Book chapter	3	28	4
Conference paper	245	1	219
Review	10	1	5
**Keywords**	**Road Accident**	**Crash Severity**	**AutoML**
Keywords Plus (ID)	2486	435	3650
Author’s Keywords (DE)	1243	201	1129
AUTHORS	Road Accident	Crash Severity	AutoML
Authors	1512	185	2150
Author Appearances	1679	199	2457
Authors of single-authored documents	12	24	24
Authors of multi-authored documents	1500	161	2126
**Authors Collaboration**	**Road Accident**	**Crash Severity**	**AutoML**
Documents per Author	0.299	0.362	0.215
Authors per Document	3.35	2.76	4.65
Co-Authors per Documents	3.71	2.97	5.32
Collaboration Index	3.42	3.74	4.89

**Table 2:** Most cited journals in the fields of road accidents, crash severity, and AutoML.

Most Cited Sources	Articles
Accident Analysis And Prevention	1463
IEEE Transactions On Intelligent Transportation Systems	239
Transportation Research Record	108
Safety Science	87
Machine Learning	62
IEEE Access	57
Journal Of Safety Research	50
Analytic Methods In Accident Research	45
Traffic Injury Prevention	28

**Table 3:** Most cited articles.

Papers	Year	Citations
[42]	2000	483
[41]	2003	323
[43]	2002	395
[48]	2010	248
[49]	2018	152
[50]	2011	137
[55]	2016	135
[51]	2019	132
[52]	2015	104
[56]	2018	101
[25]	2017	92
[53]	2014	83
[54]	2020	67

**Table 4:** Machine learning methods commonly used in CSP.

Reference	Year	Methods
[65]	2020	Multi-layer perceptron (MLP), rule induction (PART) and classification and regression trees (SimpleCart)
[57]	2020	Random forest (RF) and bayesian additive regression trees (BART)
[59]	2020	Feed-forward neural networks (FNN), support vector machine (SVM), fuzzy C-means clustering based feed-forward neural network (FNN-FCM), and fuzzy c-means based support vector machine (SVM-FCM).
[62]	2020	Naïve Bayesian (NB), Decision Tree (DT), Logistic Regression (LR), Light-GBM, and Random Forest (RF) model are proposed.
[60]	2020	Multinomial logit, mixed multinomial logit, and support vector machine (SVM)
[66]	2020	Random forest (RF), artificial neural network, and decision tree (DT)
[64]	2020	Multi-layer Perceptron (MLP), Decision Tree (DT), Random Forest (RF) classifier and Naive Bayes (NB).
[63]	2019	Random forest (RF), Adaboost with decision tree, gradient boosting decision tree (GBDT), and extreme gradient boosting decision tree (XGboost).
[58]	2019	Decision Tree (DT), K-Nearest Neighbors (KNN), Naïve Bayes (NV) and AdaBoost
[47]	2018	K-Nearest Neighbor(KNN), Decision Tree (DT), Random Forest (RF) and Support Vector Machine (SVM)
[25]	2017	Multinomial Logit (MNL), Nearest Neighbor Classification (NNC), Support Vector Machines (SVM) and Random Forests (RF)
[61]	2016	Decision trees (DT), artificial neural networks, Bayesian networks, support vector machines (SVM), and regression models

**Table 5:** Raw Data Information.

City	Records	Year	Data Source
Bogotá	66,329	2015–2019	datosabiertos.bogota.gov.co (accessed on 29 October 2021)
Medellín	150,646	2014–2018	medata.gov.co (accessed on 29 October 2021)
Bucaramanga	32,857	2012–2020	observatorio.bucaramanga.gov.co/index.php/datos-abiertos/ (accessed on 29 October 2021)

**Table 6:** Medellín case study: Description of Binary datasets.

Dataset	Instances	Distribution of Classes		Imbalance Ratios (A/B)
		People Injured (A)	Only Material Damages (B)	
Med2014	41,776	23,198	18,578	1.25
Med2015	42,427	23,550	18,877	1.25
Med2016	46,838	26,594	20,244	1.31
Med2017	42,443	22,917	19,526	1.17
Med2018	46,655	24,247	22,408	1.08

**Table 7:** Bogotá (Bog) and Bucaramanga (Buc) case studies: Description of Multiclass datasets.

Dataset	Instances	Distribution of Classes			Imbalance Ratios
		People Injured (A)	Casualties (B)	Only Material Damages (C)	
Bog2015	31,341	10,738	529	20,074	C/A = 1.87 C/B = 37.95
Bog2016	34,988	10,578	567	23,843	C/A = 2.25 C/B = 42.05
Bog2017	35,171	10,381	538	24,252	C/A = 2.34 C/B = 45.08
Bog2018	36,953	12,609	500	23,844	C/A = 1.89 C/B = 47.69
Bog2019	34,990	12,371	492	22,127	C/A = 1.79 C/B = 44.97
Buc2012	4343	1587	64	2692	C/A = 1.70 C/B = 42.06
Buc2013	4055	1519	67	2469	C/A = 1.63 C/B = 36.85
Buc2014	3723	1617	37	2069	C/A = 1.28 C/B = 55.92
Buc2015	3765	1705	47	2013	C/A = 1.18 C/B = 42.83
Buc2016	3733	1705	64	1964	C/A = 1.15 C/B = 30.69
Buc2017	3807	1903	39	1865	A/B = 1.02 A/C = 48.79
Buc2018	3910	2100	40	1770	A/B = 1.19 A/C = 52.5
Buc2019	3724	1993	42	1689	A/B = 1.18 A/C = 47.45
Buc2020	1797	1000	38	759	A/B = 1.32 A/C = 26.31

**Table 8:** Friedman’s average ranking and *p*-values obtained via the Holm post-hoc test using *CatB* and *Ag60m* as control methods in binary and multiclass datasets, respectively.

Binary Problems			Multiclass Problems		
Methods	Av. Ranking	*p*-Values	Methods	Av. Ranking	*p*-Values
**CatB**	**4**	-	**Ag60m**	**3.6786**	-
LGBM	4.2	1	Ag150m	3.8929	1
Ag150m	4.3	1	Ag15m	4.0714	1
Ag15m	4.7	1	As150m	5	1
Ag60m	4.8	1	CatB	5.2143	1
GB	5.2	1	GB	5.2143	1
Tp	5.2	1	Tp	5.4286	1
As150m	7.8	1	As60m	6.1429	1
Tuned_RF	8.4	0.958359	*As15m*	8.7143	**0.023123**
As60m	9.2	0.593928	*LGBM*	9.4286	**0.006696**
RF	10.6	0.196244	*tuned_RF*	9.4286	**0.006696**
As15m	11	0.146612	*RF*	11.7857	**0.000018**
ExtraT	11.6	0.086515	*ExtraT*	13.4286	**0**
*GNB*	14	**0.00529**	*DT*	14.1786	**0**
*DT*	15	**0.001409**	*GNB*	14.3929	**0**

## Data Availability

All data analyzed during this study are included in this article.
